# Cover crops and drought: Maize ecophysiology and yield dataset

**DOI:** 10.1016/j.dib.2021.106856

**Published:** 2021-02-09

**Authors:** Mitchell C. Hunter, Armen R. Kemanian, David A. Mortensen

**Affiliations:** a American Farmland Trust, 411 Borlaug Hall, 1991 Upper Buford Circle, St. Paul, MN 55108, United States; b 116 Agricultural Sciences and Industries Building, The Pennsylvania State University, University Park, PA 16802, United States; c Department of Agriculture, Nutrition, and Food Systems, The University of New Hampshire, Kendall Hall, 129 Main St, Durham, NH 03824, United States

**Keywords:** Cover crop, Maize, Drought, Rain exclusion, Ecophysiology, Organic agriculture

## Abstract

This dataset supports the research paper “Cover crop effects on maize drought stress and yield” by Hunter et al. [1]. Data is provided on ecophysiological and yield measurements of maize grown following five functionally diverse cover crop treatments. The experiment was conducted in Pennsylvania, USA from 2013–2015 with organic management. Cover crops were planted in August after winter wheat harvest. Cover crops were terminated in late May of the following year, manure was applied, and both were incorporated with full inversion tillage prior to planting maize. The five cover crop treatments included a tilled fallow control, medium red clover, cereal rye, forage radish, and a 3-species mixture of medium red clover, cereal rye, and Austrian winter pea. Drought was imposed with rain exclusion shelters starting in early July. Results are provided for two subplots per cover crop treatment representing ambient and drought conditions. The dataset includes: 1) soil moisture in spring and during the maize growing season; 2) maize height, leaf chlorophyll content, leaf area index, stomatal conductance, and pre-dawn leaf xylem water potential; 3) maize yield and yield components including kernel biomass, total biomass, harvest index, number of plants per subplot, ears per plant, kernel mass, and kernel number per ear, per plant, and per subplot; 4) modeled season-long radiation interception and radiation use efficiency of biomass production; and 5) maize rooting density by depth in one year only. Data was collected in the field and lab using ecophysiological instruments (e.g., SPAD meter, ceptometer, porometer, and pressure chamber). Biomass samples were taken to determine yield. Data presented have been averaged to the subplot level (ambient and drought). This dataset can inform future research focused on using cover crops and other cultural practices to improve climate adaptation in cropping systems and also may be useful for meta-analyses.

## Specifications Table

**Table:** 

Subject	Agronomy and Crop Science
Specific subject area	Drought simulation, Climate adaptation, Cover crop effects on maize physiology, Organic systems experiment, Plant ecophysiology
Type of data	Tables Figures
How data were acquired	Field- and laboratory-based measurements: • Soil volumetric moisture content using time-domain reflectometry with a TDR100 (Campbell Scientific, Logan, UT) and hand-built TDR probes equipped with three 10 cm-long stainless-steel waveguides • Maize height using a measuring stick • Leaf chlorophyll content with a SPAD meter (SPAD 500, Konica Minolta, Tokyo, Japan) • Leaf area index using a Decagon AccuPar LP-80 Ceptometer (Meter, Pullman, WA) • Stomatal conductance using a Decagon SC-1 Leaf Porometer (Meter, Pullman, WA) • Pre-dawn leaf xylem water potential using a pressure chamber (PMS Instruments, Albany, OR) • Maize yield and yield components with hand sampling, drying, and weighing • Maize rooting density by depth with the root intercept method; pits were dug with a backhoe and photographs were taken with a digital camera and metal frame Daily solar radiation data used to calculate radiation interception and radiation use efficiency was retrieved from Phase 2 of the North American Land Data Assimilation System [2]
Data format	Raw Analyzed
Parameters for data collection	Organic systems experiment with full-inversion tillage and the following rotation: winter wheat—cover crop—silage maize—cover crop—soybean. Randomized complete block design sampled in 2014 and 2015. Focus on maize following five functionally diverse cover crop treatments. Drought imposed in early July with rain exclusion shelters. Paired moisture treatment subplots: ambient and drought.
Description of data collection	Most ecophysiological measurements were collected in the field during the growing season, including soil volumetric water content and maize height, chlorophyll content, leaf area index, and stomatal conductance. Leaf water potential readings were conducted in the lab with samples transported from the field. Maize biomass samples were collected in the field and dried, weighed, and counted in the lab. Maize rooting density was assessed following maize harvest using root pit photographs; root intersections in the photographs were counted on a computer screen.
Data source location	The Pennsylvania State University Russell E. Larson Agricultural Research Center Rock Springs, Pennsylvania United States of America Latitude: 40°43′N Longitude: 77°56′W
Data accessibility	Repository name: Mendeley Data Data identification number: 10.17632/hg46dkxvd7.1 Direct URL to data: http://dx.doi.org/10.17632/hg46dkxvd7.1
Related research article	[1] M. Hunter, A. Kemanian, D. Mortensen. Cover crop effects on maize drought stress and yield. Ag. Ecosys. Env. In Press

## Value of the Data

• This is a unique and comprehensive dataset documenting short-term maize response to imposed drought conditions and functionally diverse cover crops. It can inform efforts to use cover crops and other cultural practices to improve climate adaptation in cropping systems.
• The data will be useful for agronomists and agroecologists interested in drought adaptation and cover crops.
• The data can inform the design of precipitation manipulation experiments and help generate hypotheses about the effects of cover crops on maize drought response.
• Future researchers may be able to implement more efficient and effective field research after reviewing how the many ecophysiological variables measured here responded to drought and cover crops.
• The data may also be useful in meta-analyses once there is a larger body of field experiments documenting the effects of drought and cover crops on maize ecophysiology.

## 1 Data Description

**Fig. 1:**
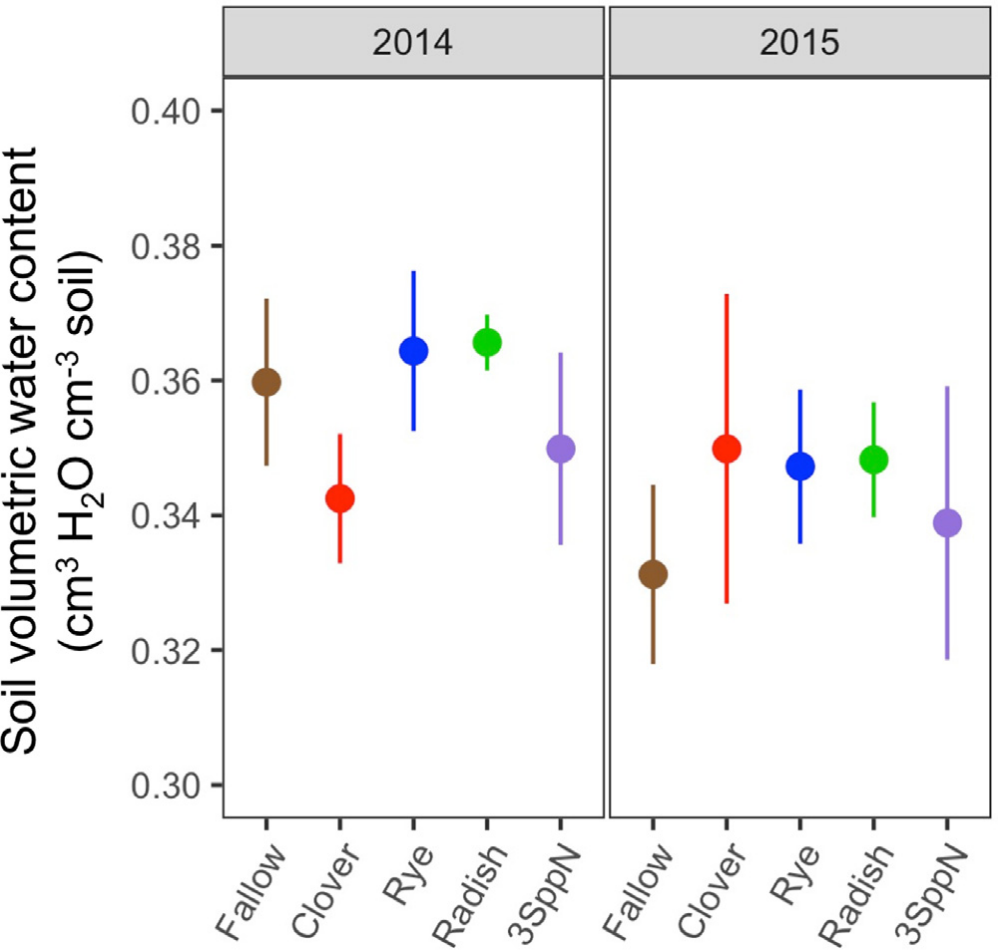
Soil volumetric water content at 20 cm depth (mean and standard error) just prior to cover crop termination (May 2, 2014 and April 29, 2015).

**Fig. 2:**
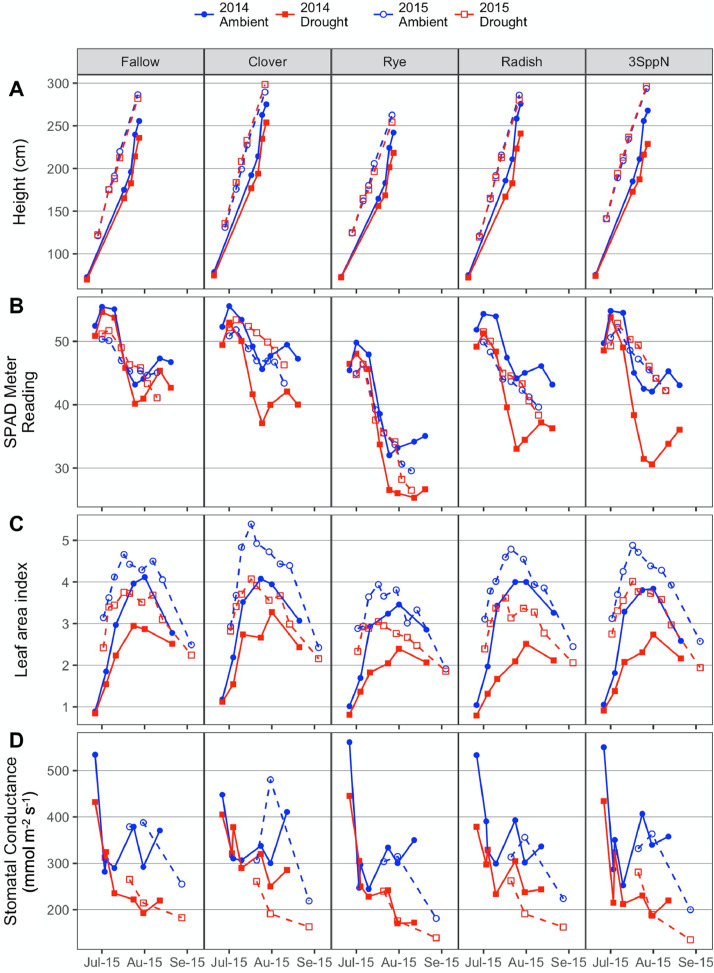
Maize (A) height, (B) SPAD meter reading, (C) leaf area index, and (D) stomatal conductance by cover crop, drought treatment, and year. Circles represent the mean value; error bars are not shown to simplify the plots. Horizontal axis shows the date in month-date format.

**Fig. 3:**
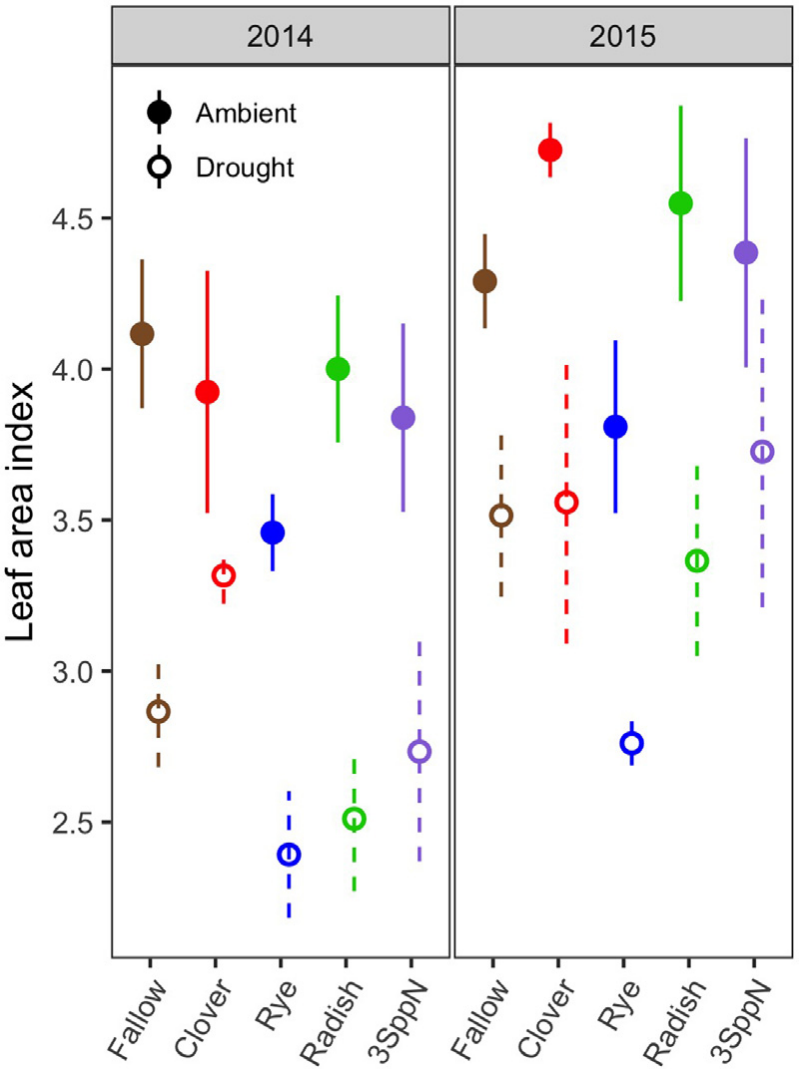
Hemispherical leaf area index (LAI) by cover crop, drought treatment, and year (mean and standard error) on a representative date during the height of the drought stress (August 15, 2014 and August 13, 2015).

**Fig. 4:**
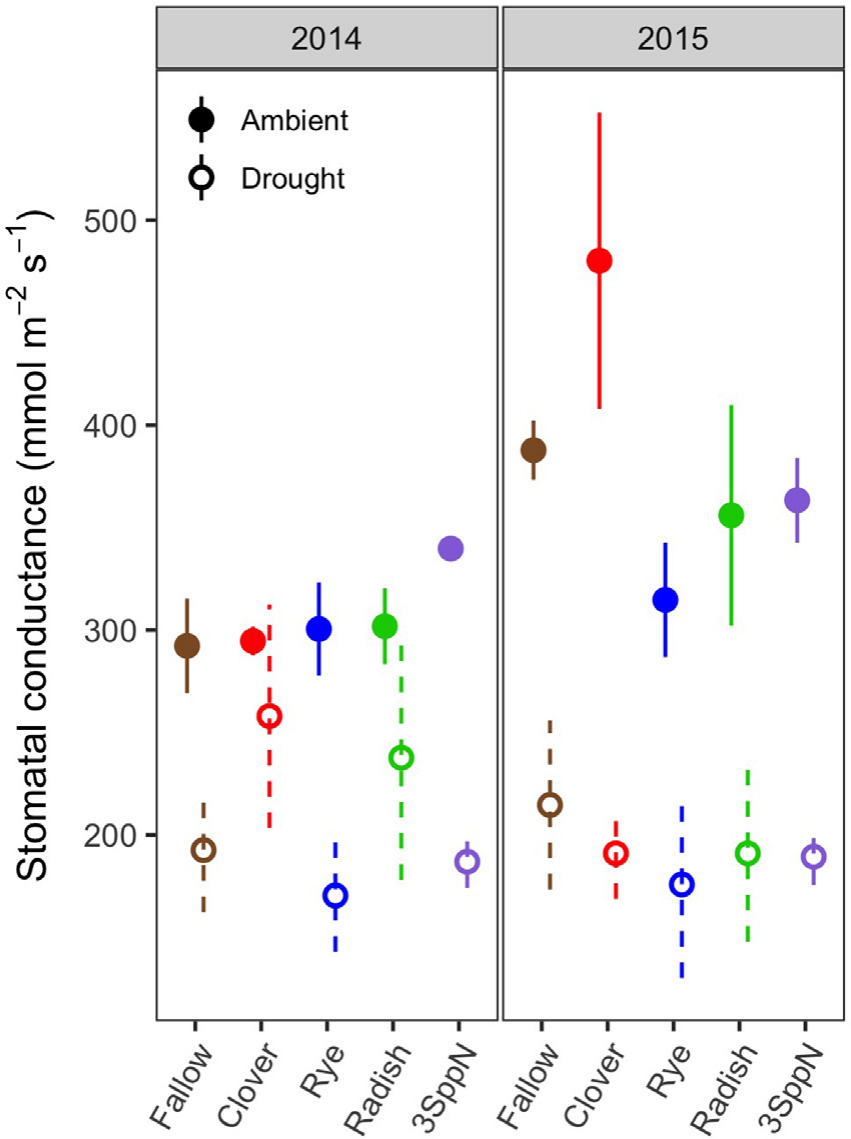
Leaf stomatal conductance by cover crop, drought treatment, and year (mean and standard error) on a representative date during the height of the drought stress (readings taken on August 14, 2014 and August 14, 2015).

**Fig. 5:**
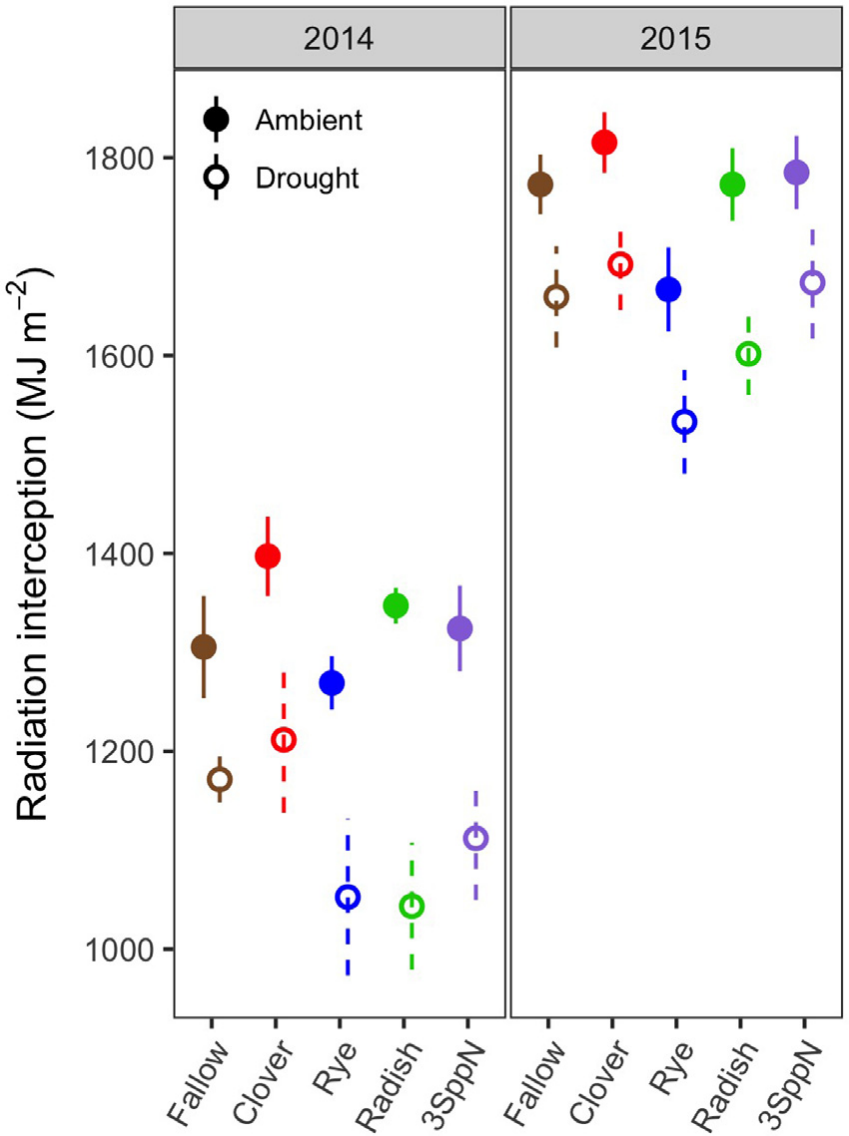
Seasonal solar radiation interception by cover crop, drought treatment, and year (mean and standard error).

**Fig. 6:**
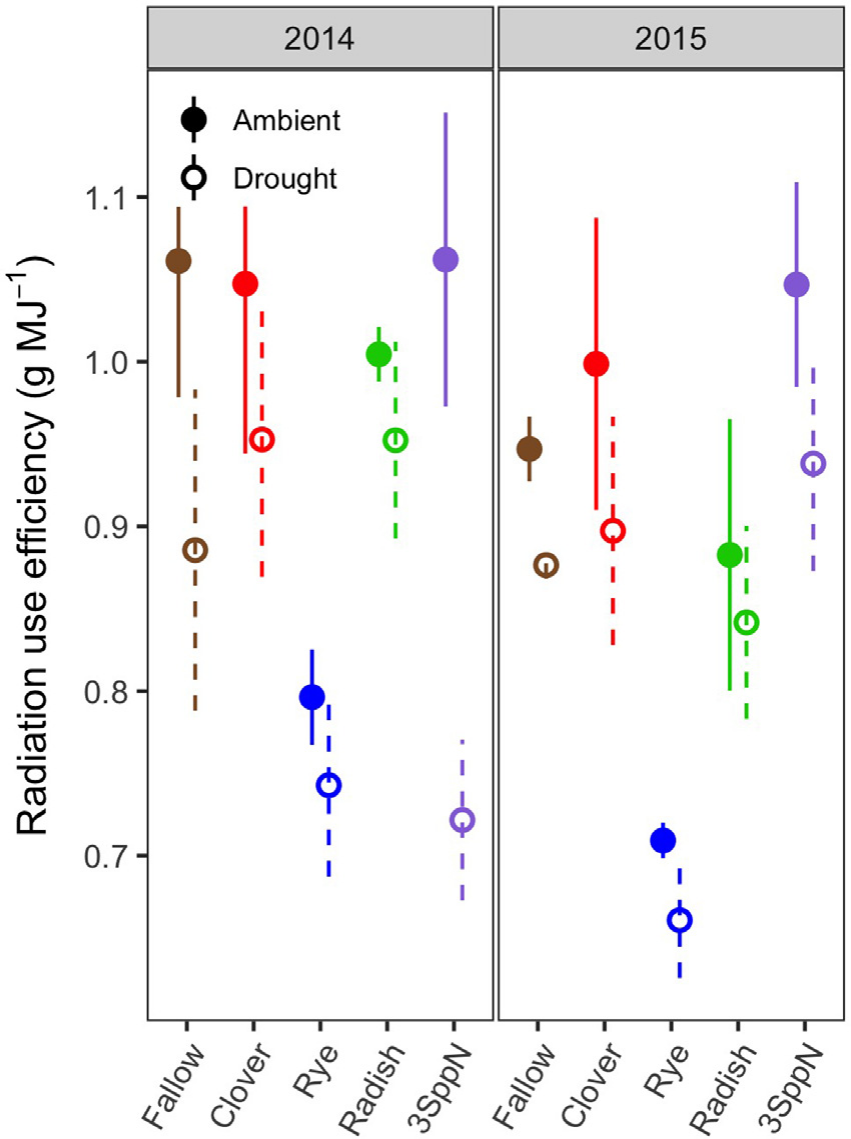
Maize radiation use efficiency (g biomass MJ^−1^ solar radiation) by cover crop, drought treatment, and year (mean and standard error).

**Fig. 7:**
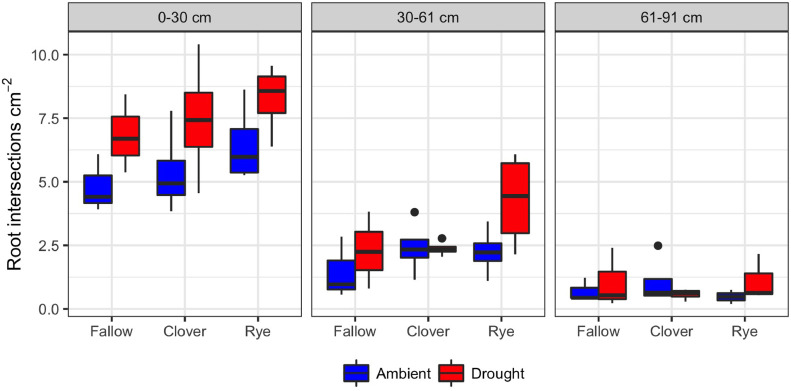
Boxplots of rooting density (root intersections cm^−2^ of vertical soil face) under the maize row, by cover crop and drought treatment. Root pits were dug following maize harvest in 2015 (10/6–10/15).

These data support the research article, “Cover crop effects on maize drought stress and yield,” by Hunter et al. [1]. The dataset includes all ecophysiological measurements taken to document the effects of cover crops and imposed drought on the maize phytometer crop. The dates on which the measurements were taken are reported in Table S1. The dataset includes two sets of soil volumetric water content measurements: 1) spring soil moisture measured at 20 cm depth during cover crop growth (Table S2; Fig. 1) and 2) growing season soil moisture measured at 10, 20, and 40 cm depths under both drought and ambient conditions (Table S3). The dataset also includes a number of maize ecophysiology measurements taken on multiple dates during the growing season: 1) height (Table S4, Fig. 2); 2) soil plant analysis development (SPAD) chlorophyll meter readings (Table S5, Fig. 2); 3) leaf area index (LAI; Table S6, Figs. 2 and 3); 4) stomatal conductance (Table S7; Figs. 2 and 4); 5) maize pre-dawn leaf xylem water potential (Table S8); 6) maize yield and yield components: kernel biomass, total biomass, harvest index, number of plants per subplot, ears per plant, kernel mass, and kernel number per ear, per plant, and per subplot (Table S9); 7) season-long radiation interception and radiation use efficiency of biomass production (Table S5, Figs. 5 and 6); and 8) rooting density by depth for the second year of the study only (2015) in select cover crop treatments (Table S10, Fig. 7).

Hunter et al. [1] contains additional figures depicting growing season soil moisture, leaf water potential, the final SPAD reading, maize kernel biomass, and the experimental setup. Hunter et al. [1] also presents data on weather; cover crop biomass and carbon-to-nitrogen ratio; soil inorganic nitrogen (N) and N mineralization rate; maize biomass N concentration; and rainout shelter microclimate, including mid-canopy air temperature, pre-dawn leaf temperature, and attenuation of photosynthetically active radiation.

The following tables are available in the dataset hosted on Mendeley Data (http://dx.doi.org/10.17632/hg46dkxvd7.1).

Table S1. Measurement dates.

Table S2. Soil volumetric water content at 20 cm depth measured on multiple dates in the spring during growth of various cover crop treatments.

Table S3. Soil volumetric water content at 10, 20, and 40 cm depths measured on multiple dates following various cover crop treatments under either ambient or imposed drought conditions.

Table S4. Maize height measured on multiple dates following various cover crop treatments under either ambient or imposed drought conditions.

Table S5. Maize soil plant analysis development (SPAD) chlorophyll meter readings taken on multiple dates following various cover crop treatments under either ambient or imposed drought conditions.

Table S6. Maize leaf area index (LAI) measured on multiple dates, season-long radiation interception, and radiation use efficiency of biomass production following various cover crop treatments under either ambient or imposed drought conditions.

Table S7. Maize stomatal conductance measured on multiple dates following various cover crop treatments under either ambient or imposed drought conditions.

Table S8. Maize pre-dawn leaf xylem water potential measured on multiple dates following various cover crop treatments under either ambient or imposed drought conditions.

Table S9. Yield and yield components of maize grown following various cover crop treatments under either ambient or imposed drought conditions.

Table S10. Density of root intersections on a vertical soil face under the maize row at the end of the growing season, at three depths, following select cover crop treatments and either ambient or imposed drought conditions.

## 2 Experimental Design, Materials and Methods

### 2.1 Experimental design

The experimental design is described comprehensively in [1] and [3]. Briefly, the study was conducted from 2013–2015 at The Pennsylvania State University Russell E. Larson Agricultural Research Center, Rock Springs, PA (40°43′N, 77°56′W) on predominantly silt-loam soils. Long-term (1980–2016) average annual precipitation at the site is 1020 mm and mean monthly temperatures range from −3 °C (January) to 22 °C (July) [2].

Cover crops were sown following winter wheat (*Triticum aestivum* L.) and before silage maize (*Zea mays* L.) in the following organically managed rotation: winter wheat—cover crop—silage maize—cover crop—soybean (*Glycine* max (L.) Merr.). Cover crop plots (24 m × 29 m) were embedded in cash crop strips (24 m × 348 m) in a randomized complete block design with four replications. The five cover crop treatments included a tilled fallow control; medium red clover (*Trifolium pratense* L. variety not stated (VNS)); cereal rye (*Secale cereale* L. cv. Aroostook); forage radish (*Raphanus sativus* L. cv. Tillage Radish); and a 3-species mixture (3SppN) of medium red clover, cereal rye, and Austrian winter pea (*Pisum sativum* L. VNS). Cover crops were established in August, terminated in May of the following year by mowing, and incorporated into the soil along with manure fertilizer with a moldboard plow.

Within each cover crop treatment, paired moisture treatment subplots (drought and ambient) were established in early July (July 8, 2014 and July 10, 2015) by installing modular rain exclusion shelters (2.7 m × 3.0 m) and demarcating a nearby, untreated area of equivalent size. To avoid edge effects, all samples were taken within a 1.2 m × 1.5 m area spanning two rows of maize at the center of the subplots [4]. Two plots in 2014 and one plot in 2015 were removed from the analysis due to persistent flooding that nullified the drought treatment.

Measurement dates for all ecophysiological measurements are presented in Table S1.

### 2.2 Soil moisture

Soil volumetric water content was measured using time-domain reflectometry (TDR; [5]) with a TDR100 (Campbell Scientific, Logan, UT) and hand-built TDR probes equipped with three 10 cm-long stainless-steel waveguides. TDR probes were tested prior to deployment.

#### 2.2.1 Spring soil moisture

Spring soil volumetric water content under the growing cover crops (Table S2) was measured with two probes per cover crop treatment installed horizontally at 20 cm depth beneath the cover crop row. Soil moisture was measured weekly from early-to-mid April until cover crop termination in early May.

#### 2.2.2 Growing season soil moisture

Soil volumetric water content during the growing season (Table S3) was measured with TDR probes installed horizontally at 10, 20, and 40 cm depths. Two sets of probes were installed in each experimental subplot, in both ambient and drought conditions. Installation holes were dug between the two middle rows of maize, with one located adjacent to each row, and waveguides were inserted toward the nearest row. Therefore, the TDR readings represent the moisture content in the rooting zone on the margin of the row and inter-row areas. TDR probes were installed following rain exclusion shelter installation and readings were taken roughly every week until harvest.

### 2.3 Maize ecophysiology

Maize height, chlorophyll content, leaf area index, stomatal conductance, and leaf water potential were measured regularly to document maize physiological responses to the cover crops and moisture treatments.

#### 2.3.1 Maize height

Following plot establishment and prior to shelter installation, the maize plants within the inner sampling area of each subplot were counted and initial height was measured on two representative plants (of median height) per subplot. Height was then measured repeatedly on the same two plants until tassels were mature; the heights of these plants were averaged to represent the whole subplot (Table S4).

#### 2.3.2 SPAD chlorophyll content

Leaf chlorophyll content (Table S7) was measured with a SPAD meter (SPAD 500, Konica Minolta, Tokyo, Japan) [6], [7]. Measurements began prior to shelter installation and continued weekly until harvest. The sensor head was placed on a clean, intact portion of a fully-expanded upper leaf at a distance of ∼20 cm from the leaf tip. One leaf on each plant within the sampling area was measured and all readings were averaged.

#### 2.3.3 Leaf area index

Hemispherical LAI (Table S5) was measured with a Decagon AccuPar LP-80 Ceptometer (Meter, Pullman, WA) with an Apogee QSO-S PAR Photon Flux ambient sensor (Apogee Instruments, Logan, UT). The ceptometer was placed on the ground beneath the maize canopy, arranged diagonally to span the row and inter-row space equally, and leveled. The ambient sensor was affixed to vertical metal conduit resting on the ground and extending above the canopy. Three randomly-chosen locations within the inner sampling area of each subplot were measured three times each and all readings were averaged. When taking readings under the rain exclusion shelters, the ambient sensor was placed under the shelter as well, so that the roof plastic impacted both sensors equally. Readings were taken within 2 h of solar noon under clear sky conditions. Leaf area index was measured weekly from mid-July through canopy closure in late August and once after senescence had begun in September.

#### 2.3.4 Stomatal conductance

Stomatal conductance (Table S6) was measured using a Decagon SC-1 Leaf Porometer (Meter, Pullman, WA). The porometer cuvette was placed on a clean, undamaged area of the leaf blade that was unshaded and oriented toward the sun [8]. This reduced variation due to irradiance and angle of incidence. At the end of the growing season, some highly stressed subplots contained few healthy leaves that were not curled, making it difficult to find leaf blades oriented toward the sun. In these cases, the best sun-lit, healthy leaf areas were chosen. Four leaves were read per subplot, two in each row, and all readings were averaged. Porometry readings began in mid-late July and continued periodically throughout the season. Readings were taken on cloud-free days to ensure that leaves were acclimated to full-sun conditions and care was taken not to shade the leaves prior to measurement. Readings were taken between 11 am and 4 pm, when transpirative demand and water stress are greatest.

#### 2.3.5 Leaf water potential

Pre-dawn leaf xylem water potential (Table S8) was measured using a pressure chamber (PMS Instruments, Albany, OR) [5]. Four samples per subplot were collected before dawn by removing the distal 15 cm of the newest fully expanded leaf. Later in the season, when newly expanded leaves were not available, healthy leaves at or above the ear leaf were chosen. Leaves were placed in a humidified plastic bag to avoid sample desiccation and the bags were placed in a cooler to maintain dark and cool conditions, then transported to the lab for reading. To prepare the leaves for reading, a cut was made with a razor blade along each side of the midrib and a clean cut was made across the end of the midrib. The midrib was inserted into the pressure chamber collar and the chamber was slowly pressurized. The pressure (bars) was recorded when xylem fluid was extruded from the cut end of the xylem. All four readings per subplot were averaged.

### 2.4 Maize yield and yield components

Maize was harvested at silage maturity (roughly 65% biomass moisture content) to align with the systems experiment in which this work was embedded, but kernel yield was assessed to provide greater insight into maize stress. Maize plants were cut by hand 15 cm above the soil surface and the ears were separated. Stalks and leaves were weighed in the field, coarsely ground using a small brush chipper, and a sub-sample was collected for subsequent analysis. Sub-samples were weighed, dried at 60 °C, and weighed again. Moisture content of sub-samples was used to calculate total shoot dry matter. Ears were removed, counted, dried at 60 °C, weighed to determine total ear dry matter, and then husks were removed. Kernels were shelled using a mechanical sheller, then weighed. Shoot and ear dry matter were summed to calculate total biomass. Harvest index was calculated as the ratio of kernel biomass to total biomass multiplied by 100. To determine mean kernel mass, 25.0 g of kernels from each subplot were counted. Due to large variation in kernel development, any kernel with yellow pericarp was included in this analysis. Mean kernel mass was then used to determine kernel number per ear, per plant, and per subplot. These measurements are presented in Table S9.

### 2.5 Radiation interception and use efficiency

The LAI readings described above were used to calculate season-long radiation interception and radiation use efficiency of biomass production (Table S5). To enable modeling of daily LAI, starting and ending dates with an LAI value of zero were added to the sequence of LAI readings. The start date was one week after planting, representing the time of emergence, and the final date was October 15th, an estimated date of full senescence, had the maize not been harvested for silage. A cubic polynomial was fit through the LAI readings for each subplot. The resulting fitted curve dipped below zero in the first two weeks; this was fixed by extrapolating linearly from a value of 0.1 on day one of the time series to the fitted value for day 14. Daily fractional interception (FI_d_) of total solar radiation was calculated for each fitted LAI value using an extinction coefficient for diffuse radiation that integrates across the range of daily solar zenith angles [9], [10], [11]. Total daily solar radiation (R_d_) was retrieved from Phase 2 of the North American Land Data Assimilation System [2]. Daily radiation interception (RI_d_) was calculated as

$$RI_{d}=FI_{d}*R_{d}$$

and season-long radiation interception (RI_s_; Table S5) was calculated for each year as the sum of all RI_d_ values up until the harvest date. Season-long radiation use efficiency of biomass production was calculated by dividing total maize biomass by RI_s_ (Table S5).

### 2.6 Rooting density

Final maize rooting density by depth (Table S10) was assessed with the root intercept method in 2015 only. Root pits were dug with a backhoe following maize harvest (October 6 to October 15). Pits were dug between the two rows of maize in the inner sampling area. Pits were one meter deep except where bedrock restricted their depth. Pit faces were parallel to and directly below the maize rows. Following excavation, the faces of the pit were lightly scraped with a shovel to remove smeared areas that would reduce visibility of intersecting roots.

Up to four faces were photographed in each pit (north and south wall, east and west side). Only clean, vertical faces directly below the maize plants were photographed; those that had caved in were ignored. Photographs were taken perpendicular to the face with a camera mounted a constant distance from a 30 cm by 30 cm metal frame. Photographs were taken at three depths: 0–30 cm, 30–61 cm, and 61–91 cm. The number of root intersections at each depth was counted on a computer screen by a single research assistant to ensure consistency.

## Ethics Statement

N/A.

## CRediT Author Statement

**Mitch Hunter:** Conceptualization, Methodology, Formal analysis, Investigation, Data curation, Writing – Original Draft, Writing – Review and Editing, Visualization, Supervision, Project Administration; **Armen Kemanian:** Methodology, Resources, Writing – Review and Editing; **David Mortensen:** Conceptualization, Methodology, Resources, Writing – Review and Editing, Supervision, Funding acquisition.

## Declaration of Competing Interest

The authors declare that they have no known competing financial interests or personal relationships which have or could be perceived to have influenced the work reported in this article.
